# Parabolic relationship between sex-specific serum high sensitive C reactive protein and non-alcoholic fatty liver disease in chinese adults: a large population-based study

**DOI:** 10.18632/oncotarget.7401

**Published:** 2016-02-15

**Authors:** Li-Ren Wang, Wen-Yue Liu, Sheng-Jie Wu, Gui-Qi Zhu, Yi-Qian Lin, Martin Braddock, Dong-Chu Zhang, Ming-Hua Zheng

**Affiliations:** ^1^ Department of Infection and Liver Diseases, Liver Research Center, The First Affiliated Hospital of Wenzhou Medical University, Wenzhou, China; ^2^ School of The First Clinical Medical Sciences, Wenzhou Medical University, Wenzhou, China; ^3^ Department of Endocrinology, The First Affiliated Hospital of Wenzhou Medical University, Wenzhou, China; ^4^ Department of Cardiovascular Medicine, The Heart Center, The First Affiliated Hospital of Wenzhou Medical University, Wenzhou, China; ^5^ Renji School of Wenzhou Medical University, Wenzhou, China; ^6^ Global Medicines Development, AstraZeneca R&D, Alderley Park, United Kingdom; ^7^ Wenzhou Medical Center, Wenzhou People's Hospital, Wenzhou, China; ^8^ Institute of Hepatology, Wenzhou Medical University, Wenzhou, China

**Keywords:** serum high sensitive C reactive protein, non-alcoholic fatty liver disease, sex-specific, risk factor

## Abstract

**Objectives:**

To evaluate the association between sex-specific serum high sensitive C reactive protein (hsCRP) levels and NAFLD in a large population-based study.

**Results:**

From Q1 to Q4, the incidence ratios were 21.1 (95% CI 17.5 24.7), 18.6 (95% CI 16.5 20.8), 24.8 (95% CI 22.4 27.2) and 31.1 (95% CI 28.5 33.6) in males and 6.2 (95% CI 4.4 8.0), 6.0 (95% CI 5.1 7.1), 11.4 (95% CI 9.2 13.7) and 19.5 (95% CI 16.1 22.9) in females. Compared with a 1.7-fold increase (Q4 vs Q2) in males, actuarial incidence increased 3.3-fold (Q4 vs Q2) in females. After adjusting for known confounding variables in this study, in the longitudinal population, compared with the reference group, those in Q1, Q3, and Q4 had HRs of 1.63 (95% CI 1.29-2.05), 1.11 (95% CI 0.93-1.31), 1.14 (95% CI 0.97-1.35) in male and 1.77 (95% CI 1.25-2.49), 1.22 (95% CI 0.93-1.59), 1.36 (95% CI 1.03-1.80) in female for NAFLD, respectively.

**Methods:**

8618 subjects from Wenzhou Medical Center of Wenzhou People's Hospital were included. Sex specific hsCRP quartiles (Q1 to Q4) were defined: 0-0.1, 0.2-0.4, 0.5-0.8 and 0.9-25.9 for male; 0-0.1, 0.2-0.6, 0.7-1.2 and1.3-28.4 for female. Applying Q2 as reference, Hazard ratios (HRs) and 95% confidence intervals (CIs) for NAFLD were calculated across each quartile of hsCRP.

**Conclusions:**

We report that a sex-specific hsCRP level is independently associated with NAFLD. The association between hsCRP and NAFLD was significantly stronger in females than in males.

## INTRODUCTION

Non-alcoholic fatty liver disease (NAFLD) is the most common form of chronic liver disease. 5% to 42% of the general population in Asian countries and 24% to 42% in Western countries suffer from the disease [1–3]. As is commonly associated with hypertension, obesity, insulin resistance, and dyslipidemia, NAFLD is the result of accumulated hepatic fat without excessive alcohol intake or other causes of liver disease [4, 5]. With NAFLD, patients have a significantly higher risk of death compared with the general population [6, 7]. A large effort has been extended to investigate risk factors associated with NAFLD and recent studies have met with some success which has encouraged further work in this field [8, 9].

High sensitive C reactive protein (hsCRP) is a biomarker of inflammation, which has been noted to predict the prevalence of type 2 diabetes, atherosclerosis and metabolic syndrome (MS) [10–12]. Though NAFLD is a pathologic condition relative to MS, whether hsCRP could function as an independent factor to predict NAFLD is poorly defined. Previously a study has suggested sex differences should be highlighted, although is not adopted in current clinical medical guidelines. Other studies have reported that compared with males, the impact of elevated hsCRP level in cardiovascular disease is likely associated with a worse prognosis in women [13–15].

In this study, we conducted our analyses in the southeast of China to evaluate the relationship between sex-specific hsCRP levels and NAFLD.

## RESULTS

### Subject characteristics

**Figure 1:**
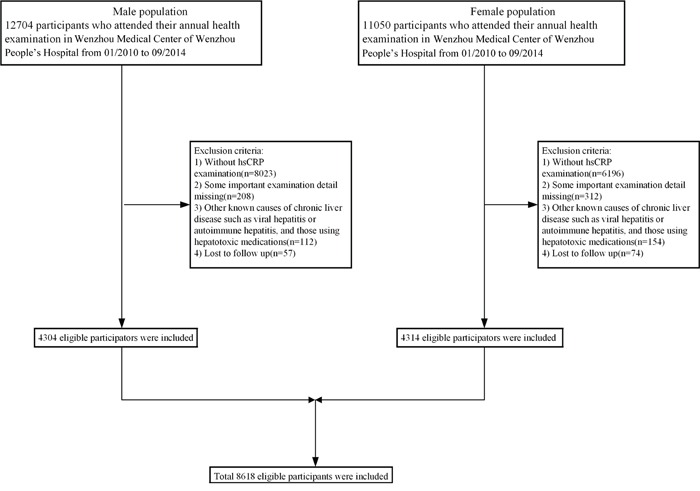
Study flow diagram A total of 23754 subjects were initially enrolled, while 15136 participants who did not meet the inclusion criteria were excluded. Finally, 60,455 individuals (49,092 in the cross-sectional population and 11,363 in the longitudinal population) were included.

A total of 23754 subjects were initially enrolled into the study, while only 8618 remained according to exclusion criteria (Figure 1). In the available data, 4304 males and 4314 females were included, with a mean age of 40.9±14.6 years and 37.2±10.5 years, respectively. The median follow-up time was 1100 days in male and 1169 days in female subjects. Baseline characteristics of study subjects are presented in Table 1 according to their quartile measurements of hsCRP. The relationship between hsCRP concentration and prevalence of NAFLD is parabolic rather than linear for extremely low or extremely high level of hsCRP, both of which resulted in higher prevalence of NAFLD. BMI, Cr, TC, TG, SBP, DBP, FPG, BUN, LDL-C were higher, whereas HDL-C was lower, in Q1 and Q4. The proportion of Q1, Q2, Q3, and Q4 in the male group is 11.7%, 29.3%, 29.1% and 29.9%. The proportion of Q1, Q2, Q3, and Q4 in the female group is 16.1%, 52.7%, 18.6% and 12.2%.

**Table 1:** Baseline characteristics of 4304 males and 4314 females, stratified by quartiles of hsCRP*

hsCRP quartiles						
	All	Q1	Q2	Q3	Q4	P value
N	4304	502	1263	1254	1285	
	4314	708	2273	804	528	
hsCRP (mg/L)	0-25.9	0-0.1	0.2-0.4	0.5-0.8	0.9-25.9	
	0-28.4	0-0.1	0.2-0.6	0.7-1.2	1.3-28.4	
Age (years)	40.92±14.55	38.20±11.32	38.63±12.39	40.81±14.29	44.35±17.02	<0.01
	37.24±10.52	34.62±8.36	36.24±9.61	40.12±11.93	40.64±12.64	<0.01
BMI (kg/m^2^	22.63±2.55	22.23±2.61	22.16±2.48	22.78±2.42	23.12±2.63	<0.01
	20.72±2.30	20.30±2.20	20.42±2.11	21.18±2.28	21.86±2.67	<0.01
Height (m)	170.33±23.85	170.73±5.56	170.34±5.64	169.90±5.82	170.58±42.78	<0.01
	159.33±5.10	159.61±4.79	159.64±4.98	158.83±5.37	158.41±5.38	<0.01
Weight (kg)	65.44±8.27	64.85±8.73	64.30±7.91	65.77±7.98	66.45±8.58	<0.01
	52.60±6.29	51.74±6.28	52.06±6.01	53.42±6.26	54.84±6.89	<0.01
SBP (mmHg)	123.92±14.44	122.34±13.42	122.02±13.28	124.13±14.23	126.18±15.75	<0.01
	112.21±13.23	110.61±12.22	111.05±12.54	114.64±13.72	115.65±15.44	<0.01
DBP (mmHg)	76.77±10.21	76.22±9.76	75.98±9.70	76.93±10.54	77.60±10.48	0.88
	69.37±9.29	68.25±8.79	68.66±8.90	71.24±9.66	71.13±10.29	0.88
HDL-C (mmol/L)	1.31±0.29	1.30±0.29	1.33±0.28	1.31±0.28	1.29±0.29	<0.01
	1.53±0.32	1.56±0.31	1.54±0.32	1.51±0.32	1.49±0.31	<0.01
LDL-C (mmol/L)	2.63±0.64	2.53±0.61	2.58±0.61	2.64±0.65	2.70±0.65	<0.01
	2.39±0.62	2.30±0.59	2.35±0.59	2.46±0.64	2.59±0.68	<0.01
TG (mmol/L)	1.44±0.91	1.36±0.84	1.33±0.74	1.45±0.88	1.55±1.09	<0.01
	0.96±0.54	0.89±0.49	0.91±0.49	1.06±0.63	1.11±0.61	<0.01
TC (mmol/L)	4.58±0.85	4.45±0.81	4.52±0.81	4.61±0.87	4.66±0.87	<0.01
	4.48±0.85	4.38±0.81	4.42±0.82	4.58±0.87	4.71±0.93	<0.01
Cr (μmol/L)	93.04±14.60	91.42±11.34	92.38±12.74	92.76±15.46	94.58±16.34	<0.01
	67.11±14.18	66.76±8.47	66.76±8.48	67.30±8.98	68.78±33.37	<0.01
UA (μmol/L)	340.39±73.78	327.43±68.63	335.21±70.50	341.21±75.75	349.70±75.74	<0.01
	225.31±53.37	220.49±53.32	222.65±52.59	230.01±52.89	236.04±55.63	<0.01
FPG (mmol/L)	5.24±0.83	5.16±0.63	5.16±0.65	5.24±0.86	5.33±1.00	<0.01
	5.01±0.47	4.97±0.43	4.98±0.45	5.07±0.51	5.06±0.52	<0.01
BUN (mmol/L)	4.63±1.24	4.55±1.20	4.54±1.14	4.64±1.17	4.75±1.39	<0.01
	4.03±1.09	3.93±1.03	3.99±1.04	4.11±1.06	4.20±1.35	<0.01
HB (g/L)	147.79±10.00	148.79±9.42	148.09±9.30	147.81±10.17	147.08±10.65	<0.01
	126.38±9.80	125.88±9.65	126.29±9.83	126.49±9.81	127.31±9.86	<0.01

BMI = body mass index, BUN = blood urea nitrogen, Cr = creatinine, DBP = diastolic blood pressure, FPG = fasting plasma glucose, HB = hemoglobin, HDL-C = high-density lipoprotein cholesterol, LDL-C= low-density lipoprotein cholesterol, SBP = systolic blood pressure, TC = total cholesterol, TG = triglyceride, UA = uric acid.

* In Table 1, the information of male was written in black and the information of female was written in red.

A parabolic relationship between hsCRP level and prevalence of NAFLD is clearly displayed in Tables 2–4. Table 2 shows that 1051 males and 377 females have developed NAFLD. From Q1 to Q4, the incidence ratios were 21.1 (95% CI 17.5 24.7), 18.6 (95% CI 16.5 20.8), 24.8 (95% CI 22.4 27.2) and 31.1 (95% CI 28.5 33.6) in males and 6.2 (95% CI 4.4 8.0), 6.0 (95% CI 5.1 7.1), 11.4 (95% CI 9.2 13.7) and 19.5 (95% CI 16.1 22.9) in females. To obtain further insight into the relationship between hsCRP level and the prevalence of NAFLD, the HRs for NAFLD were calculated after adjusting for confounding variables (Table 3 and Table 4). In male subjects using model 1, compared with the subjects in Q2, the HRs for the subjects in Q1, Q3 and Q4 were 1.70 (95% CI 1.35 2.14), 1.30 (95% CI 1.10 1.54), and 1.54 (95% CI 1.31 1.81), respectively (all P values <0.001). In female subjects using model 1, compared with the subjects in Q2, the HRs for the subjects in Q1, Q3 and Q4 were 1.59 (95% CI 1.13 2.23), 1.87 (95% CI 1.44 2.44) and 3.05 (95% CI 2.37 3.94), respectively (all P values < 0.001). Adjustment for age, BMI, SBP and DBP, using model 2, substantially attenuated the magnitude of the HRs for NAFLD when comparing the first and the forth with the second quartile of hsCRP level. Furthermore, the HRs for NAFLD were 1.63, 1.11 and 1.14 for Q1, Q3, and Q4 in males, and 1.77, 1,22 and 1,36 for Q1, Q3 and Q4, respectively, in the fully adjusted model (model 3). These results suggest that the subjects with extremely high or low hsCRP levels are more likely to develop NAFLD than individuals with moderate hsCRP levels.

**Table 2:** Adjusted hazard ratio (95% CI)* for non-alcoholic fatty liver disease according to sex specific quartiles of hsCRP

HsCRP quartiles	Number of subjects	Person-years	Number of outcomes	Incidence ratio (95%CI)*
Male				
Q1	502	1324.4	106	21.1 (17.5-24.7)
Q2	1263	3811.8	235	18.6 (16.5-20.8)
Q3	1254	3835.1	311	24.8 (22.4-27.2)
Q4	1285	4069.4	399	31.1 (28.5-33.6)
Female				
Q1	708	1985.1	44	6.2 (4.4-8.0)
Q2	2273	7416.2	138	6.0 (5.1-7.1)
Q3	804	2670.9	92	11.4 (9.2-13.7)
Q4	528	1767.5	103	19.5 (16.1-22.9)

CI: confidence incidence.

* Incidence rate of outcomes per 1,00 person-years.

**Table 3:** Association between serum hsCRP concentration and non-alcoholic fatty liver disease development in female population

hsCRP concentration quartiles				
	Q1	Q2	Q3	Q4
	708	2273	804	528
Interquartile range of hsCRP (nmol/L)	0-0.1	0.2-0.6	0.7-1.2	1.3-28.4
Median of hsCRP (nmol/L)	0.1	0.4	0.8	2.0
HR for NAFLD development (95% CI)				
Model 1	1.59 (1.13-2.23)	Ref	1.87 (1.44-2.44)	3.05 (2.37-3.94)
Model 2	1.73 (1.23-2.43)	Ref	1.31 (1.00-1.71)	1.56 (1.18-2.05)
Model 3	1.77 (1.25-2.49)	Ref	1.22 (0.93-1.59)	1.36 (1.03-1.80)

Model 1 is a univariate analysis for hsCRP. Model 2 is adjusted for age, body mass index, systolic blood pressure and diastolic blood pressure. Model 3 is adjusted for age, body mass index, systolic blood pressure, diastolic blood pressure, high-density lipoprotein cholesterol, low-density lipoprotein cholesterol, triglyceride, total cholesterol, creatinine, uric acid, fasting plasma glucose, blood urea nitrogen, hemoglobin, height and weight. CI: confidence incidence.

**Table 4:** Association between serum hsCRP concentration and non-alcoholic fatty liver disease development in male population

hsCRP concentration quartiles				
	Q1	Q2	Q3	Q4
	502	1263	1254	1285
Interquartile range of hsCRP (nmol/L)	0-0.1	0.2-0.4	0.5-0.8	0.9-25.9
Median of hsCRP (nmol/L)	0.1	0.3	0.6	1.4
HR for NAFLD development (95% CI)				
Model 1	1.70 (1.35-2.14)	Ref	1.30 (1.10-1.54)	1.54 (1.31-1.81)
Model 2	1.68 (1.33-2.12)	Ref	1.14 (0.96-1.35)	1.25 (1.06-1.48)
Model 3	1.63 (1.29-2.05)	Ref	1.11 (0.93-1.31)	1.14 (0.97-1.35)

Model 1 is a univariate analysis for hsCRP. Model 2 is adjusted for age, body mass index, systolic blood pressure and diastolic blood pressure. Model 3 is adjusted for age, body mass index, systolic blood pressure, diastolic blood pressure, high-density lipoprotein cholesterol, low-density lipoprotein cholesterol, triglyceride, total cholesterol, creatinine, uric acid, fasting plasma glucose, blood urea nitrogen, hemoglobin, height and weight. CI: confidence incidence.

**Figure 2:**
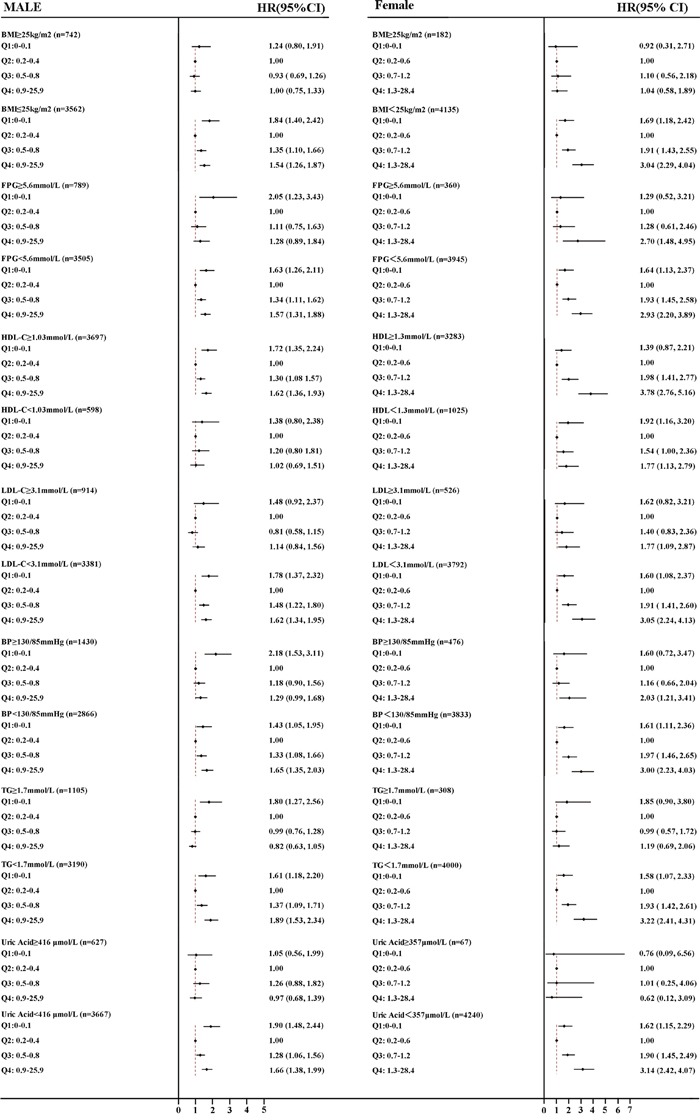
Forest plots of HRs (95% CI) for quartiles of serum high sensitive C reactive protein in the longitudinal population, stratified by sex **A.** male **B.** female. The cut-off points of BMI, FPG, HDL-C, LDL-C, BP, TG and UA were 25 kg/m^2^, 5.6mmol/L, 1.03/1.3mmol/L, 3.1mmol/L, 130/85mmHg, and 416/357μmol/L, respectively. BMI, body mass index; TG, triglyceride BP, blood pressure; HDL-C, high-density lipoprotein cholesterol; LDL-C, low-density lipoprotein cholesterol, UA, uric acid, FPG, fasting plasma glucose; HR, hazards ratio; CI, confidence interval.

Figure 2 shows the HRs for NAFLD of Q1, Q3, and Q4 using the Q2 as the reference. A stratified analysis for risk factors showed a successive increase in HRs for both males and females. Subjects had a significantly higher HR_Q1 vs Q2_ in males and HR_Q4 vs Q2_ in females in subgroups wherein BMI <25kg/m^2^, HDL-C≥1.03mmol/L or ≥1.3mmol/L (female), BP <130/85mmHg, TG<1.7mmol/L, UA<416μmol/L and FPG < 5.6mmol/L, after adjusting for all confounding variables, indicating a stronger association between hsCRP, gender and NAFLD.

**Figure 3:**
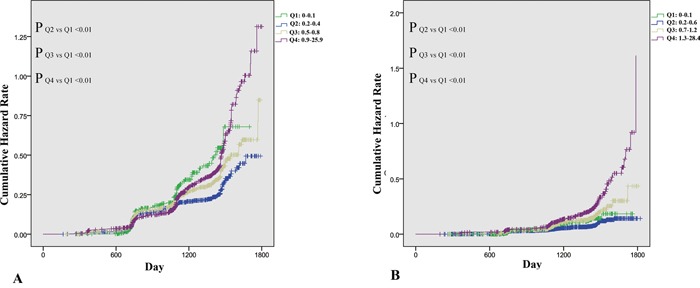
Incidence of nonalcoholic fatty liver disease (NAFLD), stratified by sex-specific quartiles of serum high sensitive C reactive protein **A.** Incidence of NAFLD in 4,304 male subjects stratified by quartiles of serum high sensitive C reactive protein. **B.** Incidence of NAFLD in 4314 female subjects stratified by quartiles of serum high sensitive C reactive protein.

Figure 3 shows the cumulative hazard rate of NAFLD, categorized by sex-specific quartiles of hsCRP. The median follow-up time was 1100 days in male and 1169 days in female. Details on the correlation of quartiles of hsCRP with incident of NAFLD are shown in Figure 3. At the time of the last follow-up, the actuarial incidence of NAFLD from Q1 to Q4 were 21.1%, 18.6%,24.8%, and 31.1% in males and 6.2%, 6.0%, 11.4%, and 19.5% in females, respectively. In Figure 3A, both extremely low (Q1) or extremely high (Q4) levels showed a difference compared with Q2, however in Figure 3B, a high level of hsCRP (Q4) was determined to be a greater risk factor for development of NAFLD. Males have a higher incidence of NAFLD, however, the hsCRP level appeared to increase the development of NAFLD in females by a factor of 3.3-fold actuarial incidence increase (Q4 vs Q2), when compared with a 1.7-fold increase (Q4 vs Q2) in males.

## DISCUSSION

This is the first and largest study specifically aimed at evaluating the association between sex-specific hsCRP level and NAFLD in representative sample of Chinese adults. In this study, we performed a prospective longitudinal population analysis that included 8618 initially NAFLD-free subjects. We present stratified data according to sex-specific quartiles and we display significant sex difference in the distribution of hsCRP.

HsCRP is an acute phase protein and it is usually used as a biomarker of inflammation. Recent studies have confirmed that the change of hsCRP level can predict the development of chronic disease. Yasuaki Hayashino demonstrated that the elevation of hsCRP appeared to make a significant contribution to an increased risk of developing type 2 diabetes or atherosclerosis while we observe a parabolic relationship between hsCRP level and the prevalence of NAFLD [10–12]. However, more than half of the hsCRP level of subjects in Yasuaki 's research was abnormal (hsCRP>3mol/L), while only 4.4 % subjects in our study had abnormal levels of hsCRP [10]. The rare incidence of abnormal hsCRP level in the general population makes it challenging to investigate the regulation of elevated hsCRP. Besides, in those studies, sex difference was not fully considered. According to previous studies and the principle of statistics, we have grouped subjects by gender [16, 17]. Our study demonstrates that associations between hsCRP levels and NAFLD may be applied to both males and females through sex-specific multivariate regression analysis. Though extremely low or high level of hsCRP all contribute to the development of NAFLD. So in addition to common concept that hsCRP lower than 3mg/L is normal, we demonstrate that extremely low hsCRP level for males and extremely low hsCRP level for females, may be associated with a higher additional risk for development of NAFLD.

One potential explanation for this association may be that the results are simply confounded by a shared background of metabolic syndrome [18, 19]. Previous research has suggested that elevation of hsCRP had strong association with development of metabolic syndrome and that in the development of NAFLD, a condition resembles metabolic syndrome, hsCRP can also be elevated for the same reason [20–22]. After adjusting for some features of metabolic syndrome and other known confounding variables, a significantly strong association between hsCRP levels and NAFLD can still be detected. The strong relationship between elevated hsCRP levels and NAFLD indicates that hsCRP overload might play some pathogenic role in the development of NAFLD. Studies have confirmed that hsCRP has a profound pro-inflammatory property [23]. In cultured human umbilical vein endothelial cells, CRP has been shown to induce the expression of ICAM-1 in a manner similar to that observed on stimulation with IL-1β [24]. The finding has been confirmed in a recent clinical research study, which included 1128 subjects from China [11].

However, previous studies have focused on the typical pathological elevated levels of hsCRP and ignored an extremely low hsCRP level. In our study, we discovered the hazard ratio in Q1 is much increased compared with Q2 in both males and females. To date, little attention has been given to discover the association between low level of hsCRP and NAFLD, we can only hypothesize that a certain hsCRP level may protect the organism from NAFLD. Previous studies have shown that CRP could activate complement through the classical activation pathway [25–27]. As the activation of the classical complement pathway by CRP is restricted to its primary stage, it is possible perhaps that CRP may function as a cyto-protective agent in the physiological stage [28]. These previous studies may, at least partially explain why the absence of hsCRP results in high prevalence of NFALD. Though previously noted, the difference in hsCRP levels between the sexes has not been widely clinically acknowledged [13]. However, in our study the difference between the incidence of NAFLD in females and elevated hsCRP was significantly greater than in males (Figure 2), after adjusting for known confounding variables. A further previous study has shown a significantly greater association between baseline CRP and cardiovascular events in females than in males [14]. Besides, in male group, not all hsCRP in Q4 is elevated, while in female group we can observe significant increase. Hormonal differences, such as estrogen functioning as an anti-inflammatory agent, may underlie the potential biochemical mechanism, however, understanding the detailed biochemistry requires further investigation in animal and cell culture models [29, 30].

Some limitations were observed in our study. The main limitation is the lack of anthropometric parameters including lifestyle, central obesity (ie, waist/hip ratio), and dietary factors, which may be helpful to better investigate the relationship between NAFLD and hsCRP levels. Further studies including more detailed personal information are required to advance our findings in this field. Secondly, the diagnosis of NAFLD in our study was based on ultrasonography, which has lower sensitivity and specificity versus liver biopsy. However, as a widely used technique in epidemiological surveys of NAFLD, liver ultrasonography has several strengths, including safety, economical, and practical utility. Thirdly, our subject health details were collected from annual health examination, thus most of our subjects hsCRP levels are normal. How typical pathologic levels of hsCRP stimulate the development of NALFD is a subject for future investigative studies. Fourthly, the accuracy of our hsCRP is 0.1mg/L. Though some articles have used other cut-off values, 0,1mg/L is currently the mostly reliable value used.

In conclusion, we have clearly demonstrated that hsCRP is a vital factor associated with the prevalence and development of NAFLD. Elevated and extremely low hsCRP levels appear to increase the prevalence and incidence of NAFLD in females rather than in males. Ultimately, the change of hsCRP may be indicative of elevated risk of NAFLD in the general population and especially in females.

## MATERIALS AND METHODS

### Study design

This study is a cohort study aimed at evaluating the prospective association between hsCRP and NAFLD. The population was conducted from 23754 initially fatty liver disease-free individuals who underwent annual health screen in Wenzhou Medical Center of Wenzhou People's Hospital. The study period was initiated in January 2010 and concluded in September 2014. Before the study was conducted, verbal informed consent was acquired from each subject and the protocol of this study was approved by the ethics committee of Wenzhou People's Hospital.

### Ultrasonography test

The diagnosis of NAFLD was made according to Guidelines for the assessment and management of nonalcoholic fatty liver disease in the Asia-Pacific region [31]. In general, NAFLD can be diagnosed when imaging tests forecast hepatic steatosis, excluding alcohol abuse, viral hepatitis, drug-induced liver disease, and autoimmune liver disease. Hepatic steatosis was defined by the presence of at least 2 of 3 abnormal detections on abdominal ultrasonography: diffusely increased echogenicity (“bright”) liver with liver echogenicity greater than kidney or spleen, vascular blurring, or deep attenuation. Two experienced imaging specialists, blind to study design, were invited to assess abdominal ultrasonography during the ultrasonic examination. If the diagnoses made by the two specialists were in disagreement or indeterminacy, a third specialist was invited.

### Data collection

All subjects were instructed to fast overnight and refrain from exercise the previous day before clinical examination and data recording was conducted in the following morning. Medical history and a health habit inventory were recorded by trained medical staff. Blood pressure (BP: SBP and DBP) were measured through noninvasive automated sphygmomanometer (OMRON, Japan) while the subjects sat in a quite environment. Normal BP range was defined less than 130/85 mmHg [3]. Height and weight were measured without shoes or outerwear. The result of weight in kilograms divided by height in meters squared was defined as body mass index (BMI), an index of body fat. Fasting blood samples were collected from antecubital vein and were later used for biochemical measurement analysis without being frozen. The experimental procedures were consistent and the laboratories were certified according to International Organization Standardization. The biochemical measurements included hsCRP, albumin, blood urea nitrogen (BUN), creatinine (Cr), fasting plasma glucose (FPG), HB (hemoglobin), uric acid (UA), total cholesterol (TC), triglyceride (TG), high-density lipoprotein cholesterol (HDL-C), and low-density lipoprotein cholesterol (LDL-C). An automated analyzer (Abbott AxSYM) was used to for all measurements employing standardized methodology. Antinuclear antibody (ANA) was assessed using indirect immunofluorescence. ELISA assays (IEGAN, Freedom evolyzer/150) were used to detect Hepatitis C and human immunodeficiency virus antibodies. Hepatitis B virus serologic markers were detected in each subject (Abbott AxSYM). Anti-liver cytosol antibody Type 1 (anti-LC-1), anti-liver/kidney microsomal antibody Type 1 (anti-LKM-1) and soluble liver antigen/liver pancreas antigen (SLA/LP) were evaluated using immunoblot analysis (Euroimmun, Lubeck, Germany). Clinical, laboratory and ultrasonography tests at follow-up were identical to those used at the start of the study.

### Exclusion criteria

Subjects meeting the following criteria were excluded: without hsCRP examination; one or more important examination detail(s) missing; any other known potential causes of chronic liver disease such as viral or autoimmune hepatitis and subjects using hepatotoxic medications, subjects who were lost to follow-up.

### Statistical analysis

In a previous study data presented in sex-specific quartiles showed a statistically significant difference in hsCRP between males and females [32]. Quartiles were classified as follows: Q1: 0-0.1, Q2: 0.2-0.4, Q3: 0.5-0.8 and Q4: 0.9-25.9 for male; Q1: 0-0.1, Q2: 0.2-0.6, Q3: 0.7-1.2 and Q4: 1.3-28.4 for female.

In this longitudinal study, hazard ratios (HRs) and 95% confidence intervals (CIs) for NAFLD were calculated based on Cox's proportional hazards regression. Kaplan-Meier analysis was used to calculate the cumulative hazard of NAFLD during the follow-up, categorized by sex-specific quartiles of hsCRP. Multivariable models were applied to adjust for confounding variables. Model 1 is a univariate analysis for hsCRP. Model 2 is adjusted for age, body mass index, systolic blood pressure and diastolic blood pressure. Model 3 is adjusted for age, body mass index, systolic blood pressure, diastolic blood pressure, high-density lipoprotein cholesterol, low-density lipoprotein cholesterol, triglyceride, total cholesterol, creatinine, uric acid, fasting plasma glucose, blood urea nitrogen, hemoglobin, height and weight.

Continuous variables were summarized as mean ± standard deviation (SD), and categorical variable are presented as counts or percentages (%). The characteristics of the study population stratified by hsCRP quartiles were compared using a 1-way analysis of variance (ANOVA) or Kruskal-Wallis test for continuous variables while *χ*^2^ test was used for assessment of categorical variables. All P values are 2-sided, a P value of <0.05 was considered statistically significant and analyses were performed in SPSS version 20.0 (SPSS, Chicago, IL)

## Abbreviations

BMI: body mass index
BUN: blood urea nitrogen
CIs: confidence intervals
Cr: creatinine
DBP: diastolic blood pressure
FPG: fasting plasma glucose
hemoglobin: HB
HDL-C: high-density lipoprotein cholesterol
HRs: hazard ratios
hsCRP: high sensitive C reactive protein
LDL-C: low-density lipoprotein cholesterol
NAFLD: non-alcoholic fatty liver disease
UA: uric acid
SBP: systolic blood pressure
TC: total cholesterol
TG: triglyceride
